# Lymph node swelling combined with temporary effector T cell retention aids T cell response in a model of adaptive immunity

**DOI:** 10.1098/rsif.2021.0464

**Published:** 2021-12-01

**Authors:** Sarah C. Johnson, Jennifer Frattolin, Lowell T. Edgar, Mohammad Jafarnejad, James E. Moore Jr

**Affiliations:** ^1^ Department of Bioengineering, Imperial College London, London, UK; ^2^ Department of Biomedical Engineering, Johns Hopkins University School of Medicine, Baltimore, MD, USA

**Keywords:** lymph node, effector T cells, agent-based model, adaptive immunity, T cell dynamics

## Abstract

Swelling of lymph nodes (LNs) is commonly observed during the adaptive immune response, yet the impact on T cell (TC) trafficking and subsequent immune response is not well known. To better understand the effect of macro-scale alterations, we developed an agent-based model of the LN paracortex, describing the TC proliferative response to antigen-presenting dendritic cells alongside inflammation-driven and swelling-induced changes in TC recruitment and egress, while also incorporating regulation of the expression of egress-modulating TC receptor sphingosine-1-phosphate receptor-1. Analysis of the effector TC response under varying swelling conditions showed that swelling consistently aided TC activation. However, subsequent effector CD8^+^ TC production was reduced in scenarios where swelling occurred too early in the TC proliferative phase or when TC cognate frequency was low due to increased opportunity for TC exit. Temporarily extending retention of newly differentiated effector TCs, mediated by sphingosine-1-phosphate receptor-1 expression, mitigated any negative effects of swelling by allowing facilitation of activation to outweigh increased access to exit areas. These results suggest that targeting temporary effector TC retention and egress associated with swelling offers new ways to modulate effector TC responses in, for example, immuno-suppressed patients and to optimize of vaccine design.

## 1. Introduction

The lymphatic system is a network of organs and lymphatic vessels that maintains fluid balance and delivers crucial antigen information to lymph nodes (LNs) for adaptive immunity initiation. LNs contain compartments populated by T cells (TCs), B cells, fibroreticular cells (FRCs) and lymphatic endothelial cells (LECs) [1,2]. When antigens are presented (either suspended in lymph or captured by incoming antigen-presenting cells such as dendritic cells (DCs)), the LNs' physical environment changes. Swelling of LNs is a well-known consequence of antigen presentation, but the effects of swelling on processes crucial for adaptive immunity are not well understood.

TCs and B cells mainly enter LNs by transmigrating from blood vessels in the paracortex, while lymph-borne DCs migrate into the paracortex across the sub-capsular sinus (SCS) floor [3,4]. Typically, one in 10 000 naive TCs express a complementary TC receptor to the antigen fragment presented by DCs within a major histocompatibility complex class I (MHCI) (to CD8^+^ TCs) or class II (MHCII) (CD4^+^ TCs) molecule [5,6]. With sufficient affinity and stimuli, TCs undergo activation, secrete inflammatory and activation-facilitating cytokines and differentiate into effector and memory TCs [7].

The mechanisms driving LN swelling include DC presence, B cell signalling and trapping of non-activated TCs [8–11]. Regardless of the trigger, within 2 days, the TC exit rate drops (LN shutdown), blood flow to the LN increases and inflammatory signalling results in a three- to fivefold increase in TC recruitment via high endothelial venules (HEVs) [12–15]. From 48–96 h, LN mass increases two- to fivefold, accompanied by a similar increase in cellularity, and FRCs elongate to accommodate LN size increase [11,16,17]. Subsequent LEC and FRC proliferation allows maintenance of LN architecture during further expansion [10,17,18]. The LN blood vessels also grow, increasing blood vessel volume roughly proportional to overall LN volume, accompanied by further TC recruitment [9,14,19].

Between 2 and 5 days after immunization, the antigen-presenting DC (agDC) number in the LNs peaks, TC activation and proliferation is underway and TC egress increases three- to sixfold [10,11,20,21]. The expansion of medullary and SCS areas aids increased TC egress [22]. Recruitment of TCs then declines, HEV, FRC and TC proliferation subsides, remaining effector TCs may undergo apoptosis and LNs return to baseline volume while memory cells recirculate [19].

Throughout these processes, TC egress is modulated by sphingosine-1-phosphate-1 receptor (S1P_1_r) expression and chemokine signalling axes. After entering the LN, TCs express S1P_1_r at low levels but begin S1P_1_r re-expression after 2 h [23,24]. TCs exit LNs by probing and subsequently entering cortical sinuses in the paracortex or the medullary interface, aided by chemotaxis [25,26]. During inflammation, TC S1P_1_r expression is reciprocally regulated by CD69, an early TC activation marker. This mechanism contributes to the initial decrease in TC egress, termed LN shutdown, and later to the specific retention of activated TCs [15,27]. Differentiated effector TCs re-express S1P_1_r, facilitating egress [28].

The ability to investigate the importance of LN swelling in these processes is limited experimentally by a lack of means to modulate swelling without interfering directly with other aspects of adaptive immunity. We chose to develop an agent-based model (ABM) that could describe macro-scale geometric changes, micro-scale TC and DC interactions and capture emergent behaviour by modelling the probabilistic behaviour of thousands of cells. Beyond the desire for a better understanding, we aim to provide a means for designing experiments that explore potential therapeutic means of modulating LN swelling.

Fixed-volume ABMs have provided insight into interactions relevant to vaccine design; for example, the effects of antigenic peptide separation on TC activation, influential aspects of TC–DC interaction and memory TC production [29–33]. An ABM to investigate chemotactic influence included a form of paracortical expansion, where grid compartment number remained equal to TC number and exit portal number altered to maintain a mean TC residence time. This model suggested that the relative chemokine level is important but may underestimate changes in crowding and egress with swelling [34–36]. Simulations integrating a fixed-volume lattice-based model and a continuous model of chemokine diffusion showed that early antigen removal and TC exit regulation affected the balanced system dynamics, indicating that macro-scale swelling is likely to significantly affect micro-scale TC activity [37].

In summary, the careful trafficking and coordination of immune cell movements in the LNs suggest that LN swelling may significantly impact the adaptive response. We developed a computational ABM to investigate this hypothesis. The results suggest an important role for regulating early effector TC retention to maintain the benefits of LN swelling on overall effector TC response.

## 2. Material and methods

### 2.1. Agent-based model geometry

**Figure 1.**
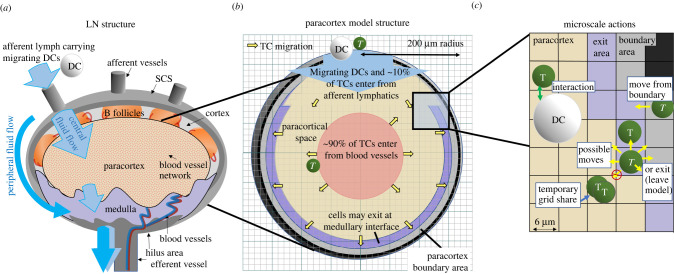
Model geometry and structure. (*a*) LN structure displaying arriving lymph containing agDCs. (*b*) TCs enter in the centre of the spherical paracortical model and exit near the interface with the medulla and SCS. The paracortex radius expands as a function of TCs present. (*c*) TCs move to adjacent grid compartments, interact with neighbouring agents and are influenced by grid compartment properties, which are updated each time step.

We aimed to replicate a murine LN by integrating experimentally obtained parameters. The paracortex was modelled as a sphere with initial radius *R*_0_ = 200 µm, derived from confocal images of murine LNs [2,38]. Geometric symmetry was assumed so that one-half of the total spherical geometry was modelled. The modelling domain was divided into cuboid grid compartments, with edge length 6 μm (figure 1*c*). For each grid compartment, we tracked which region of the paracortex was represented, such as ‘exit’, ‘boundary’ or ‘outside’.

### 2.2. Modelling swelling

We collected data from murine experiments regarding change in LN mass and volume, TCs, structural cells, migrating DCs, TC recruitment and TC egress following antigenic stimulus application [9,11,12,19,20]. Based on these data (electronic supplementary material, table 1, S1 file), we calculated paracortical volume *V* at time *t* as a sigmoidal function of the number of TCs present (*N*_*t*_), constrained by maximal swelling (*V*_max_). Parameter *T*_mid_ determines the required number of TCs to reach half-maximal swelling, which we initially estimated as a doubling of the baseline number of TCs. Slope parameter (*l*) determines curve steepness and thus the rate of change in volume around *T*_mid_,

2.1 $$V(t)=\;\frac{V_{\max}}{1+e^{l(N_{T}(t)-T_{\mathrm{mid}})}}\;.$$

We applied paracortical swelling or contraction to achieve the desired volume by changing the region type that each grid compartment represented, so that the model boundaries can extend or shrink. Internal areas, such as entry and exit areas, are defined as a constant percentage of the changing outer radius (figure 1*a*; electronic supplementary material, figure A, S1 file). Initial TC increase is permitted without triggering significant swelling, reflecting initial inhibition of stromal cell proliferation by secretion of interferon type 1 [39]. A delayed volume increase in response to TC number is in agreement with the cell signalling switch at day 2 to favour LN expansion, through mechanisms such as increased elasticity of the FRC network and LEC proliferation [11,40].

### 2.3. T cell recruitment

Under baseline conditions, the TC recruitment rate was specified as 2000 TCs/hour, with the naive TC transit time (*T*_res_) defined to range from 6 to 24 h and a constant TC-to-compartment ratio assumed (1.2 in electronic supplementary material, S1 file). In accordance with HEV images, 90% of TCs entered at ‘entry’ compartments designated as the inner half of the paracortical radius [41]. Remaining TCs entered via the SCS interface.

When calculating the TC recruitment rate (*T*_in_), acute TC recruitment changes due to inflammation-induced signalling cascades at the HEVs were incorporated using the inflammatory index, *I*_F_. This index affects TC influx when antigenic presence *D* (sum of MHCII, equation (2.3)) rises above threshold *T*1, which is the minimal DC number required to elicit a response [30]. The value of *I*_F_ increases proportionally with antigenic presence by a recruitment factor (*R*_F_) up to a maximum inflammation-induced TC recruitment, threshold *T*2 (equation (2.4)). The volume of the entry grids is representative of blood vessel volume (*V*_B_), which changes proportionally with paracortical volume. We assumed that the TC recruitment rate (*T*_in_) is additionally influenced proportionally by ${\bar{V}}_{B}$ (equation (2.2)), based on correlation of the blood vessel network length with LN volume [9,42]. TC influx was therefore defined as

2.2 $$T_{\mathrm{in}}(t)=\;\frac{N_{T}}{T_{\mathrm{res}}}\;I_{F}(t){\bar{V}}_{B}(t),$$

where *N_T_* is the initial TC number, *T*_res_ is the naive TC transit time, *I_F_* is the inflammatory index and ${\bar{V}}_{B}$ is the normalized blood vessel volume. Default egress parameters were selected to maintain equilibrium between TC entry and egress at baseline; therefore, *N_t_*, LN volume and, consequently, *T*_in_ remained stable in the absence of antigenic stimulation. Threshold values for *T1*, *T2* and *R_F_* were estimated from initial TC recruitment rate changes due to inflammation, while considering changes due to HEV growth and agDC number present [11–15,20,43]. The inflammatory index *I*_F_ was calculated as

2.3 $$D=\overset{N_{\mathrm{DC}}}{\underset{n=1}{\sum }}MHC_{II}(t)$$

and

2.4 $$I_{F}(t)=\{\begin{array}{cc} 1 & D\leq T1\\ 1+R_{F}D & T1<D\;\leq T2\\ 1+R_{F}T2 & D>T2, \end{array}\;$$

where *N*_DC_ is the number of agDCs present, *D* is the sum of MHCII carried by each agDC and Recruitment Factor *R*_F_ is an estimated increase in recruitment rate.

### 2.4. T cell egress and S1P_1_r expression

Relative TC expression of S1P_1_r (SP) is designated a default value of 1 and overall probability of TC egress (*E*) when entering an exit area is defined as *E* = *P_e_*.*SP*, where *P_e_* was experimentally determined to maintain influx and egress equilibrium under non-inflammatory conditions. We altered SP under three conditions (electronic supplementary material, figure B in S1 file). Following TC entry into the paracortex S1P_1_r remained downregulated (SP_in_ = 0.1) for 45–180 min, before re-expressing due to low paracortical S1P concentration [24]. An ‘LN shutdown’ mechanism was included by downregulating S1P_1_r (SP_inflam_ = 0.4) on all TCs when sufficient antigenic presence (summation of MHCII) was detected, estimated to correspond to 6 h post-agDC appearance. Activation-induced TC S1P_1_r downregulation was represented by decreasing S1P_1_r expression 10-fold when TCs initially activated (SP_act_ = 0.01), increasing S1P_1_r expression as TCs differentiated into early effector TCs (SP_early_ = 0.4) and further increasing expression when effector TCs underwent eight or more divisions (SP_late_ = 1) [22,44,45].

### 2.5. T cell and dendritic cell motility and interaction

TCs were modelled as spheres of volume 150 μm^3^ that initially occupied 55% of the total paracortex volume, approximately 5 × 10^4^ TCs in our hemispheric model [46]. The frequency of antigen-specific (cognate) TCs (*F*_cog_) was derived from *in vivo* reports with default 1 × 10^−4^, resulting in approximately five cognate TCs at initiation [6]. DCs were modelled as 6 μm radius spheres and interacted with TCs within a two-grid radius, up to a maximum number of TCs at once (*B*_max_). The total number of DCs is calculated as a proportion of TCs (*ϕ_DC_*), with a default value of 0.04 (approx. 2500 DCs). Each agDC presented a decaying MHC signal, and during interactions cognate TCs gained ‘stimulation’ (S) at rate *κ*s, proportional to MHCs presented, while losing stimulation at rate *λ*S. Similar to previous models, the probability of TC activation and, after a minimum of four proliferations, differentiation into effector or memory TCs was determined as a sigmoidal function of accumulated stimulation [31,35,47]. See electronic supplementary material, S1 file for full rules.

### 2.6. Computation

**Figure 2.**
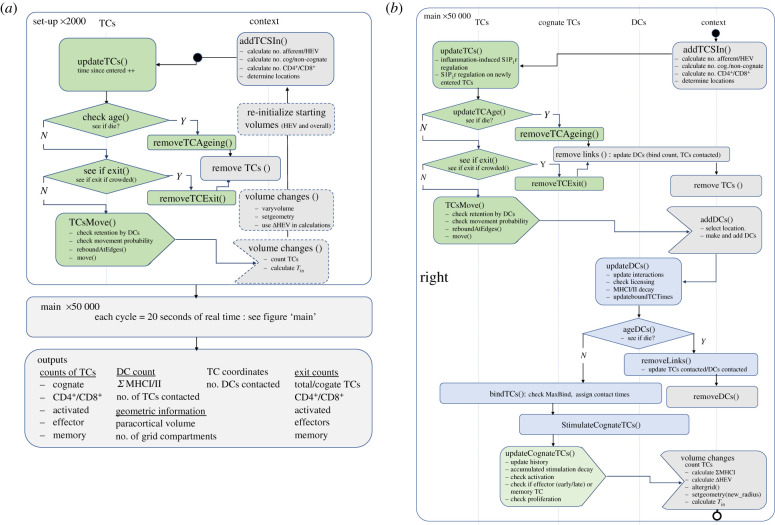
Structure of the model code. (*a*) The model is initiated in the absence of stimulus, capacity for paracortical volume change is then introduced and variables storing starting volumes are updated. Agents represent cells (TCs and DCs), store the interaction history and present state information. The ‘context’ describes the environment and ‘projections’ between agents allow information transfer. Each time step represents 20 s. (*b*) Following equilibration, the ‘main’ function calls a repeated series of sub-functions (see electronic supplementary material, S2 file) describing DC arrival and TC response, updating properties (electronic supplementary material, figure C in S2 file) each time step.

We built a class-based ABM (electronic supplementary material, figure C in S1 file) in Java using RepastSimphony (repast.sourceforge.net) with repeated rules each time step (figure 2). Further descriptions are in the electronic supplementary material, S2 file. We carried out batch simulations on the Imperial College High Performance Computing cluster and analysed data in Matlab. Model code is available on GitHub at https://github.com/johnsara04/paracortex_model_johnson19.

### 2.7. Parameter selection and sensitivity analysis

We estimated our parameters from published studies with inflammation-induced mice or previous relevant models (electronic supplementary material, table A in S1 file). To ensure awareness of influential but uncertain or biologically unconstrained parameters, we carried out a global sensitivity analysis. We used Latin Hypercube sampling to select 300 parameter combinations, simulated each set three times and recorded the TC number (activated, effector, memory, effector exited and memory exited). Partial rank correlation coefficients (PRCCs) were calculated between each parameter and output for each day (3–13), assuming monotonic relationships [48]. We report significant PRCCs with a strength greater than 0.2 (electronic supplementary material, S4 file).

### 2.8. Validation and model robustness

To ensure that we did not overfit the model to one swelling scenario, we simulated four experiments that mimic *in vivo* and/or *in vitro* experiments, holding our parameter selection constant, aside from a single parameter. In each scenario, we compared the effects on TC activation and CD4^+^ and CD8^+^ effector TC response with relevant published studies. We inhibited S1P_1_r downregulation on activated TCs as carried out by Gräler *et al.* [49] and Lo *et al.* [24]. We varied the initial proportion of cognate TCs, as carried out by Moon *et al.* [50] and Obar *et al.* [51]. We varied the agDC number, as carried out by Kaech *et al.* [52] and Martín-Fontecha *et al.* [53], and we simulated early DC apoptosis, as carried out by Prlic *et al.* [54].

## 3. Results

### 3.1. The model produces realistic baseline T cell motility and response to agDCs

**Figure 3.**
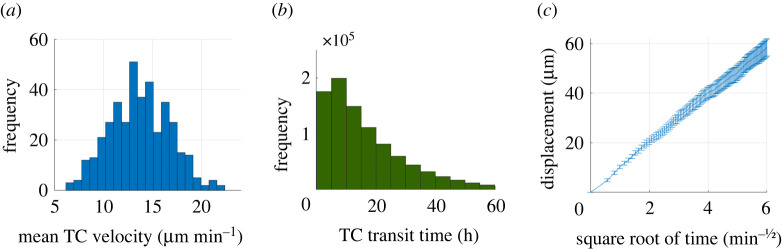
Baseline TC motility (*n* = 200). (*a*) Mean TC velocity. (*b*) Most TCs transit in less than 24 h. (*c*) Mean (±s.e.m.) of TC displacement showed a linear relationship to the square root of time, indicating random walk behaviour.

We confirmed that the calibrated model produced an average TC velocity (*n* = 200) of 13.1 µm min^−1^, reaching up to 24 µm min^−1^ (figure 3*a*), in line with murine *in vivo* measurements [41,55–58]. The mean TC paracortex transit time was 13.1 h (*n* = 16 000), ranging from 20 min to greater than 60 h (figure 3*b*), in line with observations that 74% of CD4^+^ TCs and 64% of CD8^+^ TCs transit murine LNs within a day [59]. The linear relationship between TC displacement and square root of time (figure 3*c*) illustrated the maintenance of random walk behaviour [60]. The motility coefficient (CM) was 63.2 µm^2^ min^−1^, which is within the 50–100 µm^2^ min^−1^ range observed in mice [61].

**Figure 4.**
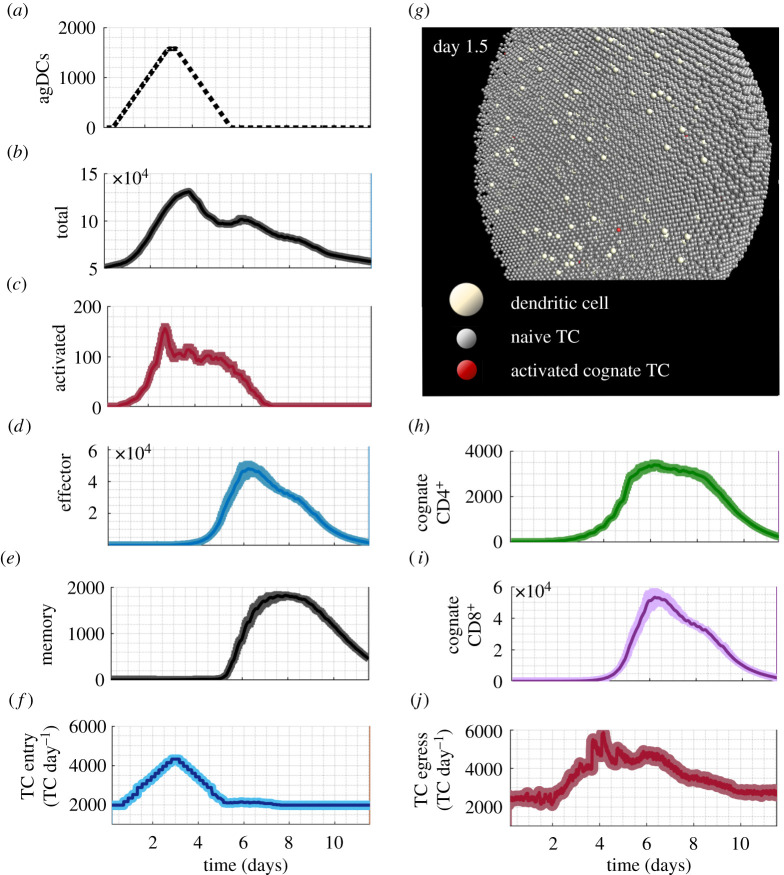
TC responses in the paracortex following entry of agDCs under baseline conditions. The average result with s.e.m. of 12 simulations. (*a*) Incoming agDCs. (*b*) The total number of TCs peaked at 3.5 days, comprising mainly non-cognate naive TCs. (*c*) Activated TC appearance began 12 h after the first agDCs entered. (*d*) Effector TC number peaked at day 6. (*e*) Memory TCs appeared at 5 days and 25% of the peak number remained at the simulation end. (*f*) TC entry rate increased twofold, peaking at day 3. (*g*) Model interface showing day 1.5 with agDCs present and TC activation initiated. (*h*) Cognate CD4^+^ TCs began extensive proliferation at day 2.2. (*i*) Cognate CD8^+^ TCs began proliferation at day 4 and reached numbers 10-fold more than cognate CD4^+^ TCs. (*j*) TC egress rate declined between day 1 and 2, then increased threefold by day 4.

TC responses to agDC stimuli corresponded well to data from *in vivo* trials in mice, sheep and rats, displaying the expected phases of TC trafficking and response (electronic supplementary material, figure A in S3 file). TC numbers began to increase approximately 6 h after initial agDC entry, and by day 11 had returned to within 15% of pre-stimulus values (figure 4*b*), in line with temporal responses observed *in vivo* [11,12,20]. The appearance of activated, effector and memory TCs began at 16–24 h, day 3.5 and day 5 post-agDC entry, respectively, in agreement with *in vivo* reports and cell-culture models [62,63]. Effector CD4^+^ TCs appeared 1–1.5 h before CD8^+^ effector TCs (figure 4*h,i*). As observed *in vivo*, the peak cognate CD8^+^ TC number was an order of magnitude higher than that of CD4^+^ TCs [64,65]. The contraction phase began at day 7 and continued through day 11 (figure 4*b*). An increase in TC egress rate peaked a day later than the increase in TC entry rate (figure 4*f*,*j*), corresponding well with *in vivo* observations [16,66].

### 3.2. Model robustness

**Figure 5.**
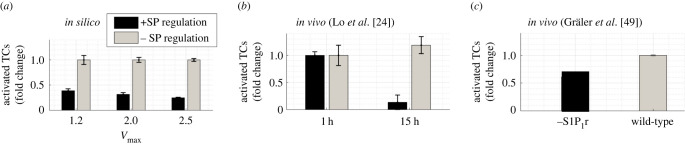
Comparing model predictions with reported *in vivo* effects of S1P_1_r downregulation on TC activation. (*a*) Simulation results (*n* = 10) with and without S1P_1_r downregulation (±SP regulation) showed total activated TC number reduced by 60%, 72% and 81% (mean±s.e.m.) at *V*_max_ = 1.2, 1.5 and 2. (*b*) Pre-activated TCs over-expressing S1P_1_r (−SP regulation) were transferred into mice. Retention of activated TCs 15 h later fell by 90% compared with transferred wild-type TCs (SP+ regulation). Adapted from Lo *et al.* [24]. (*c*) In mice with constitutive S1P_1_r expression, activated TC number in LNs 24 h post-immunization dropped by 40%. Adapted from Gräler *et al.* [49].

Holding the default parameters and varying a single parameter at a time to mimic *in vivo* and *in vitro* experiments resulted in reasonable TC behaviour. For example, preventing S1P_1_r downregulation post-antigenic stimulus detection *in silico* reduced activated TC number by 60–81% (figure 5*a*). A study transferring activated TCs that over-express S1P_1_r into mice LNs, removing S1P_1_r-mediated retention, resulted in 90% less activated TC retention than in control mice when measured 15 h later (figure 5*b* [24]). A study using mice with constitutive TC expression of S1P_1_r showed a 40% reduction in activated TCs post immunization (figure 5*c*) [49]. See electronic supplementary material, S3 file for a comparison of varying cognate frequency, agDC presence and duration of stimuli application.

The global parameter sensitivity analysis indicated that the dominant parameters in determining the target outcomes of TC activation, total TC effectors and TCs exited were F_cog_, TDC_in_ and *V*_max_. The unconstrained parameters used to describe signal integration and parameterize activation or differentiation probability curves were not identified as significantly influential in determining target outcomes (*p* > 0.05, *R^2^* < 0.2) (electronic supplementary material, figure A and tables A–C in S4 file).

### 3.3. Paracortical swelling consistently aids T cell activation

**Figure 6.**
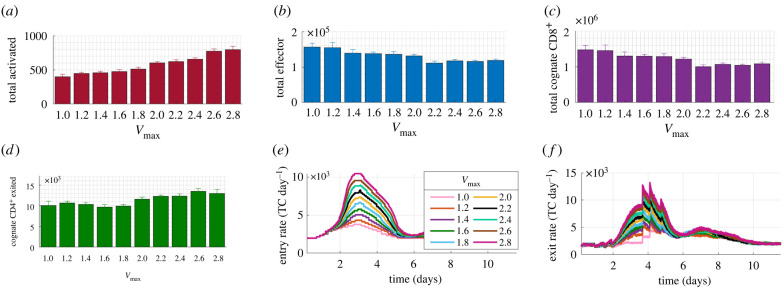
Changes in TC response in the paracortex when varying swelling. Between *V*_max_ = 1 and 2.8, (*a*) total activated TCs doubled and positively correlated with *V*_max_ (*R*^2^ = 0.96, *p* = 1.07 × 10^−6^), (*b*) total effector TCs decreased 0.3-fold, negatively correlating with *V*_max_ (*R*^2^ = 0.86, *p* = 1.23 × 10^−4^), (*c*) total cognate CD8^+^ TCs negatively correlated with *V*_max_ (*R*^2^ = 0.855, *p* = 1.28 × 10^−4^), and (*d*) total cognate CD4^+^ TCs that exited increased 1.3-fold, positively correlating with *V*_max_ (*R*^2^ = 0.76, *p* = 0.001). (*e*,*f*) Peak entry and exit rate increased proportionally to *V*_max_. Results are the mean of *n* ≥ 7 simulations with s.e.m. displayed.

When maximal swelling (*V*_max_) was varied from 1 to 2.8, the activated TC number doubled and positively correlated with *V*_max_ (*R*^2^ = 0.96, *p* < 10^−5^) (figure 6*a*). The total number of effector TCs decreased by 15% (figure 6*b*) and negatively correlated with *V*_max_ (*R*^2^ = 0.86, *p* < 10^−3^) but the number of effector TCs that exited by day 10 did not significantly vary (electronic supplementary material, figure E in S3 file).

Assessment of TC subgroups showed that the total cognate CD8^+^ TCs present decreased by 25% (figure 6*c*), negatively correlating with *V*_max_ (*R*^2^ = 0.855, *p* < 10^−3^) but there was no change in the number of exiting cognate CD8^+^ TCs (electronic supplementary material, figure E in S3 file). Conversely, the number of cognate CD4^+^ TCs that left the paracortex by day 10 increased by 30% and positively correlated with *V*_max_ (*R^2^* = 0.76, *p* = 0.001) (figure 6*d*) but cognate CD4^+^ TCs present did not vary significantly (electronic supplementary material, figure E in S3 file).

The peak TC recruitment rate positively correlated with *V*_max_, meaning that the absolute number of cognate TCs entering increased with swelling (figure 6*e*). TC egress rate increased with *V*_max_ from day 3 to day 6 (figure 6*f*). Increased TC activation but decreased effector TC number remained when LN volume increased as a linear function of TCs (electronic supplementary material, figure A in S5 file).

### 3.4. Reduced effector T cell response with swelling was not due to a lack of agDC availability

**Figure 7.**
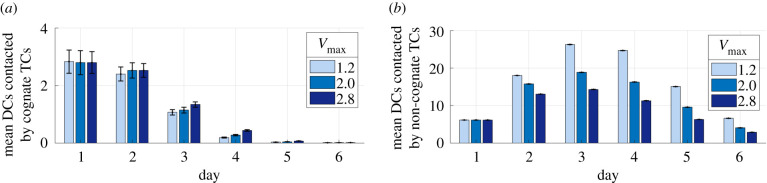
Changes in DC and TC contact with swelling from day 1 to day 6. With increased swelling, the mean number of DCs contacted by (*a*) cognate TCs did not decrease but (*b*) non-cognate TCs contacted fewer DCs.

We then analysed the mean number of interactions with DCs by cognate and non-cognate TCs present each day from day 1 to day 6 at different maximal swelling (*V*_max_ = 1.20, 2.0 and 2.5). We found that there was no decrease in the mean number of agDCs that each cognate TC contacted on all days (figure 7*a*). We also found a slight increase in the number of contacts by day 3, a time point that corresponds with peak swelling. The mean number of short contacts by non-cognate TCs decreases with swelling (figure 7*b*). These results suggest that there is no decrease in the availability of DCs to cognate cells with swelling.

### 3.5. Paracortical swelling can hinder effector T cell production in some circumstances

**Figure 8.**
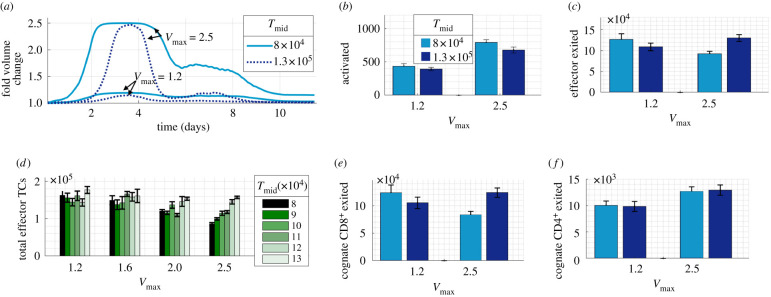
Varying the pattern of paracortical swelling. (*a*) The paracortex swells earlier and for a longer duration with a low *T*_mid_. (*b*) Increased swelling aided TC activation. (*c*) With a lower *T*_mid_, as swelling increased, the number of effector TCs that exited decreased. (*d*) Further simulations varying *T*_mid_ confirmed that, at a large swelling (*V*_max_ = 2.5), delayed swelling with a higher *T*_mid_ resulted in more total effectors TCs (*R*^2^ = 0.97, *p* = 3 × 10^−4^). (*e*) This effect was due to altered CD8^+^ TC number as (*f*) CD4^+^ TC number increased with *V*_max_ but was unaffected by varying *T*_mid_.

We carried out simulations with a small or large maximal swelling (*V*_max_ = 1.2 or 2.5) while applying a lower (8 × 10^4^ TCs) or higher (13 × 10^4^ TCs) *T*_mid_, making swelling occur relatively earlier or later (figure 8*a*). Regardless of *T*_mid_ value, at least 40% more activated TCs were recorded with a large *V*_max_ compared with a small *V*_max_ (figure 8*b*). With an earlier (low *T*_mid_) and larger swelling, the total number of effector TCs and effector TCs exited dropped significantly (*p* < 0.05) (figure 8*c*). However, with later swelling (high *T*_mid_), a larger swelling no longer reduced effector TC number. This altered effector TC response was due to a change in cognate CD8^+^ TC number, which showed the same pattern of results (figure 8*e*). There was no change associated with *T*_mid_ in cognate CD4^+^ TC response (figure 8*f*). Varying maximal swelling and *T*_mid_ over a wider range showed that the positive correlation of *T*_mid_ with effector TCs exited was only significant with a larger swelling (*V*_max_ = 2.5) (figure 8*d*), likely due to the greater impact of varying *T*_mid_ with larger swelling (figure 8*a*).

### 3.6. S1P_1_r-mediated temporary retention of early effector T cells increased T cell response

**Figure 9.**
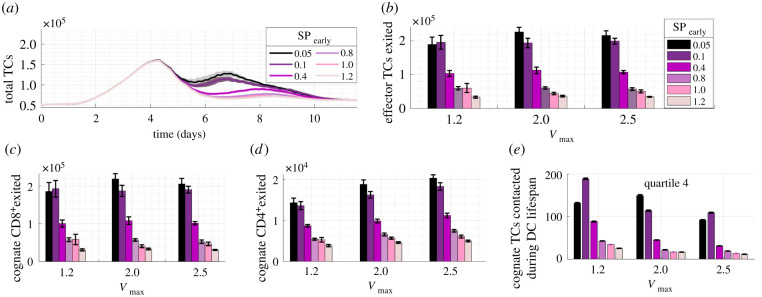
Temporary retention of effector TCs by modulating S1P_1_r expression on newly differentiated TCs (SP_early_). (*a*) Reducing SP_early_ resulted in higher total TC number. (*b*) Number of effector TCs exited was affected more by SP_early_ than by *V*_max_, negatively correlating with SP_early_ *(R*^2^ > = 0.92, *p* < 0.005). SP_early_ negatively correlated with (*c*) CD8^+^ TCs exited (*R*^2^ > 0.92, *p* < 0.005) and (*d*) CD4^+^ TCs exited (*R*^2^ > 0.91, *p* < 0.005). (*e*). The mean number of cognate TCs contacted increased as SP_early_ was lowered to 0.1 at each *V*_max_ but overall decreased with *V*_max_.

When we increased S1P_1_r downregulation by lowering SP_early_ from the estimated default value of 0.4, a sustained increase in total TCs resulted, despite the action acting on early effector TCs only (figure 9*a*). Unlike during simulations with default SP_early_ (figure 6), effector TC number did not decrease with swelling. Instead, when SP_early_ was lowered from 0.4 to 0.1, approximately 15% and 10% more effector TCs were produced with larger *V*_max_ of 2.0 and 2.5, respectively. At every maximal swelling value, SP_early_ inversely correlated with effector TC number (*R^2^* = 0.92, 0.93, 0.92, *p* < 0.005). Reducing SP_early_ from 0.4 to 0.05 doubled the number of effector TCs exiting and increasing SP_early_ to 0.8 halved the number (figure 9*b*).

When analysing the TC sub-populations, both CD4^+^ and CD8^+^ effector TCs that exited the paracortex by day 10 doubled when SP_early_ was decreased from 0.4 to 0.05 (figure 9*c*,*d*). This indicated that CD4^+^ TCs do maintain further proliferative capacity in the model.

The number of TCs contacted by DCs increased as SP_early_ was decreased, but overall decreased with swelling; therefore, this was not a driving factor of increased effector TC number (figure 9*e*). Implementation of an alternative model with non-specific constraint of TC egress by reducing expansion in the exit area also resulted in increased effector TC exit but produced unrealistic prolonged swelling above a 1.4-fold swelling (electronic supplementary material, figure B in S5 file).

### 3.7. Non-specific early LN shutdown with a doubling of LN volume did not significantly impact effector T cell production

**Figure 10.**
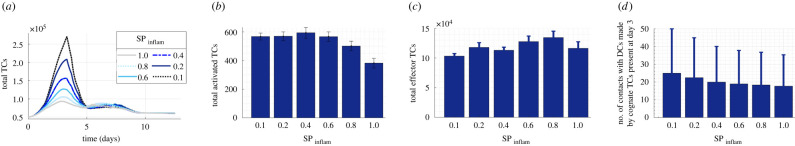
Varying LN shutdown by modulating initial inflammation-induced S1P_1_r downregulation (SP_inflam_). Modulating S1P_1_r expression from 0% to 90% downregulation (SP_inflam_ = 1 to SP_inflam_ = 0.1). (*a*) Total TC number decreases several fold but (*b*) TC activation increases (*R*^2^ = 0.83, *p* = 0.01) while (*c*) effector TC production shows no trend. (*d*) Non-cognate TCs contact more DCs as SP_inflam_ is increased (*R*^2^ = 0.93, *p* = 1.7 × 10^−3^).

We also varied the degree of initial LN shutdown, by varying SP_inflam_ from 0.1 (90% downregulation) to SP_inflam_ = 1 (no shutdown). We permitted a doubling of LN volume. Increasing non-specific S1P_1_r downregulation from 60% to 90% resulted in a sharp, threefold higher peak in the total number of TCs (figure 10*a*), which is less physiologically realistic than with our default parameters. As SP_inflam_ decreased, TC activation increased (*R^2^* = 0.83, *p* = 0.01) (figure 10*b*), but no trend with total effector TCs was identified (figure 10*c*). We found no correlation between increased LN shutdown and the mean number of contacts with DCs by cognate TCs present at day 3 but a positive correlation with DCs contacted by non-cognate TCs (*R^2^* = 0.93, *p* = 0.0017) (figure 10*d*).

### 3.8. Boosting T cell response when cognate T cell frequency is low

**Figure 11.**
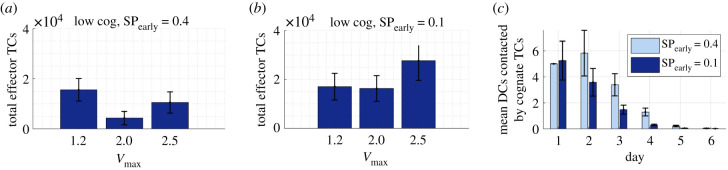
Swelling combined with increased retention of early effector TCs can improve response. With a 10-fold lower cognate frequency of 10^5^ and (*a*) default estimated S1P_1_r expression compared with (*b*) increased early effector S1P_1_r downregulation. (*c*) The increased response is not due to increased DC access (*V*_max_ = 2.0).

Simulations using a 10-fold lower cognate TC frequency showed a larger decrease in effector TC number with swelling than the simulations with default cognition. With lower cognition, we observed a mean 73% fall with a twofold swelling, compared with a mean 17% decrease with 10-fold higher cognition (figure 6*b*). With *V*_max_ = 2.5, we recorded a mean 33% fall compared with a 5% decrease with 10-fold higher cognition (electronic supplementary material, figure E-vii in S3 file). We repeated the simulations with increased early effector TC S1P_1_r downregulation (SP_early_ = 0.1). This resulted in swelling of 2.0- or 2.5-fold benefiting the response. Assessment of TC and DC interactions showed that this was not due to an increase in contact with DCs (figure 11*c*).

## 4. Discussion

In this work, we aimed to better understand the effects of LN swelling in the formation of TC responses and identify key features that can influence TC behaviour. Our study builds on work using ABMs to investigate the impact of signal integration kinetics, TC migration and interaction dynamics on TC response with a focus on macro-scale alterations and accompanying changes in egress and recruitment [29–31,33,35].

We found that permitting LN swelling consistently aids TC activation but allowing increased swelling can inhibit subsequent effector TC response if it resulted in increased opportunity for effector TCs to egress prior to optimal proliferation. Our modelling rules meant that LN swelling contributes to increased TC recruitment in a positive feedback loop and therefore to a higher absolute number of cognate TCs entering into the paracortex, increasing TC activation probability, in agreement with *in vivo* TC recruitment studies [14]. In our model, the swelling also presented a greater number of exit points and therefore increased the opportunity for effector TC egress, counteracting increased TC recruitment. A change in contact between TCs and cognate TCs was not a driving factor.

A key finding was that temporary S1P_1_r-mediated retention of newly differentiated effector TCs increased effector TC production in scenarios where effectors egress prior to reaching sufficient proliferation. The increased production was not due to increased contact with DCs (figure 9*e*), and non-specific TC retention in the first few days had no impact on effector TC response (figure 10). Swelling also increased effector TC production when the exit area growth with swelling was constrained in alternative models (electronic supplementary material, figure B in S5 file).

We also found that, with a low TC cognition rate, temporary S1P_1_r-mediated retention of newly differentiated effector TCs doubled effector TC response when combined with swelling, but swelling alone negatively impacted response. Here, swelling increases initial TC recruitment and the initial number of cognate TCs, but must be combined with increased temporary retention of newly differentiated cells to benefit the response.

The temporary nature of this S1P_1_r modulation is crucial to increase effector TC number. Permanent inhibition of effector TC S1P_1_r expression has been carried out *in vivo*, and, therapeutically, S1P_1_r downregulation is the mechanism of multiple sclerosis drug fingolimod. This acts to indefinitely retain effector TCs in the LN to prevent an autoimmune response [67]. Temporary downregulation on selectively newly differentiated TCs may prove technically difficult, suggesting that an alternative means of retention is desirable [28].

In contrast with our results, transferring 10^6^ cognate TCs into murine LNs, while facilitating swelling by inducing FRC elongation and inhibiting FRC contraction, enhanced the subsequent TC proliferative response [17]. The authors suggest that this may be due to reduced inhibition of TC activation by FRCs, or increased DC migration. Despite omission of these features, our model is in agreement with the increased TC activation. With an inflated initial cognate TC number that exponentially proliferated, the proportional effects of TC egress may be less as the TC proliferative response is also relative to starting cognate TC frequency (electronic supplementary material, figure B in S3 file) [6,50,51]. We may also overestimate the negative effects of egress area availability with swelling, but highlighting the sensitivity of egress changes and temporary retention as a means to counteract sub-optimal responses remains an important result.

Our model contains unconstrained parameters that relate to signal gain and loss (*κ*s and *λ*), activation and differentiation probability curves (Act*_μ_*_4+_, Act*_μ_*_8+_, Dif*_μ_*_4+_, Dif*_μ_*_8+_), TC recruitment and paracortical swelling (electronic supplementary material, S4 file). The sensitivity analysis showed that the parameters populating activation and probability curves were not highly influential, and the influence of signal integration patterns has been the focus of previous modelling studies [31,35,47]. Our model was not overfitted to a single scenario, as we also compared variations in stimulus strength, duration and TC cognition rate with results from *in vivo* experiments that were not used in parameter estimation.

Limitations of our model include the lack of chemotactic influences, FRC network omission and a simplified LN geometry. We prioritized the inclusion of S1P_1_r downregulation over the role of chemokine receptor CCR7 because, when CCR7 and S1P_1_r TC expression is inhibited *in vivo*, TCs still migrate to the paracortex boundary but the lack of S1P_1_r expression prevents exit [28]. The critical influence of retention in our model suggests that future iterations should include a wider range of retentive influences. We omitted DC migration and LN-resident DCs, but our results indicate that DC availability is not a limiting factor. To model alternative stimuli, for example antigen-encoding RNA or free antigen resulting from intra-nodal vaccination, information regarding free antigen arrival rate and relative expression of MHC molecules after capture and processing by resident DCs would be required.

Several models suggest that TC contacts are not significantly influenced by FRC network inclusion and we assumed that, regardless of the underlying FRC structure, TCs migrate with a random walk [55,58,61,68–70]. When the FRC is modelled as a small-world network, damaging the network by removing 50% of nodes can significantly affect effector TC response [71]. We assumed that FRC stretch and proliferation helps to maintain FRC architecture during our modest swelling [11].

Model fidelity is also limited by a lack of information on exit point availability during swelling, but the sensitivity to alterations in egress suggests that exit area change with swelling presents as a crucial area to focus future studies. Future model iterations including features such as lymph flow and pressure alterations (along with fluid exchange with nodal blood vessels) could also significantly improve the representation of swelling, and thus TC egress and retention. It has been well established that changes in hydrostatic and oncotic pressure differences across nodal blood vessel walls can reverse the net fluid exchange [72,73]. Afferent lymphatic flow, and thus DC number, to LNs also increases with immune response, as well as influencing chemokine concentration fields and likely mechanoresponsive cell expression of signalling molecules and receptors. Furthermore, intra-nodal vaccine injection would result in a bolus of fluid. A key next step is therefore to couple the ABM to a computational flow model.

## 5. Conclusion

Our results suggest that, although permitting LN swelling aids TC activation, events that increase opportunity for TC egress prior to optimal proliferation, such as early LN swelling, inhibit effector response. We found that temporary retention of newly differentiated effector TCs boosted effector TC response. This effect is particularly of interest when the initial TC response is small, for example in immuno-suppressed patients, or desirable, such as when optimizing vaccine design to minimize antigen dose. Although permanent blockade of effector TC egress has been used clinically to treat multiple sclerosis, temporary retention of effector TCs to boost effector TC production presents a novel mechanism to enhance immune reaction. Further clinically relevant insights include identification of the importance of alterations in TC egress with swelling, implying that the manipulation of factors involved in the underlying swelling mechanisms is a worthwhile clinical strategy. Variability in response among individuals is an accepted reality in immunology, and variations in ability to produce LN swelling (a readily measurable biomarker) could provide a means to project likely immune response. Our results also highlight the influence that retentive features, including factors such as chemokines, have on effector TC response, which may be more practical clinical targets to manipulate.

## Data accessibility

Model code is available on GitHub at johnsara04/paracortex_model_johnson19. The data are provided in the electronic supplementary material [74].
